# Correlation of Particle Traversals with Clonogenic Survival Using Cell-Fluorescent Ion Track Hybrid Detector

**DOI:** 10.3389/fonc.2015.00275

**Published:** 2015-12-07

**Authors:** Ivana Dokic, Martin Niklas, Ferdinand Zimmermann, Andrea Mairani, Philipp Seidel, Damir Krunic, Oliver Jäkel, Jürgen Debus, Steffen Greilich, Amir Abdollahi

**Affiliations:** ^1^German Cancer Consortium, Translational Radiation Oncology, National Center for Tumor Diseases, German Cancer Research Center, Heidelberg University Medical School, Heidelberg, Germany; ^2^Heidelberg Ion Therapy Center, Heidelberg, Germany; ^3^Heidelberg Institute of Radiation Oncology, National Center for Radiation Research in Oncology, Heidelberg, Germany; ^4^National Center for Oncological Hadrontherapy, Pavia, Italy; ^5^Light Microscopy Facility, German Cancer Research Center, Heidelberg, Germany; ^6^Division of Medical Physics in Radiation Oncology, German Cancer Research Center, Heidelberg, Germany

**Keywords:** clonogenic survival, fluorescent nuclear track detector, carbon ion irradiation, 53BP1, DNA damage foci

## Abstract

Development of novel approaches linking the physical characteristics of particles with biological responses are of high relevance for the field of particle therapy. In radiobiology, the clonogenic survival of cells is considered the gold standard assay for the assessment of cellular sensitivity to ionizing radiation. Toward further development of next generation biodosimeters in particle therapy, cell-fluorescent ion track hybrid detector (Cell-FIT-HD) was recently engineered by our group and successfully employed to study physical particle track information in correlation with irradiation-induced DNA damage in cell nuclei. In this work, we investigated the feasibility of Cell-FIT-HD as a tool to study the effects of clinical beams on cellular clonogenic survival. Tumor cells were grown on the fluorescent nuclear track detector as cell culture, mimicking the standard procedures for clonogenic assay. Cell-FIT-HD was used to detect the spatial distribution of particle tracks within colony-initiating cells. The physical data were associated with radiation-induced foci as surrogates for DNA double-strand breaks, the hallmark of radiation-induced cell lethality. Long-term cell fate was monitored to determine the ability of cells to form colonies. We report the first successful detection of particle traversal within colony-initiating cells at subcellular resolution using Cell-FIT-HD.

## Introduction

Radiotherapy with protons and heavier ions has become a swiftly growing field, and it is becoming an integrative part of therapy of solid tumors, due to its high success rate in treating certain tumors (1). Nevertheless, intracellular molecular events caused by interactions between the charged particles and cellular structures are not yet well understood. Development of novel approaches that will facilitate deciphering those processes is of high relevance for the field.

Recently, a cell-fluorescent ion track hybrid detector (Cell-FIT-HD) was engineered by our group. It provides information on spatial correlation between single ion traversals and the events within a cell (2, 3). Cell-FIT-HD technology is based on growing a cellular monolayer (biological compartment) on a surface of a fluorescent nuclear track detector [FNTD; physical compartment (4)]. Due to its unique design, Cell-FIT-HD enables simultaneous investigation of microscopic beam parameters and their effect on various cellular structures and biological processes, using confocal laser scanning microscope (5).

In this work, we investigated the feasibility of Cell-FIT-HD for colony formation analysis. Colony formation assay (also called clonogenic assay), developed in 1950s (6), is the most reliable and relevant method for studying the efficacy of the radiation treatment *in vitro*. It has been named “gold standard” in radiation research as it combines contribution of all types of cell death, as well as ability of surviving cells’ to indefinitely proliferate and form colonies (7, 8). For particle therapy planning, clonogenic survival data are of utmost importance for studying radiobiological effectiveness (RBE) and they continue to be used as the main biological experimental outcome for testing biophysical models for predicting tumor response to irradiation (9). Colony formation and cellular clonogenic survival after irradiation are highly depend on radiation potential to induce complex, difficult to repair, DNA damage [such as DNA double-strand breaks (DSB)] (10). Commonly used molecular surrogate for detecting DNA damage and DNA DSB is p53 binding protein 1 (53BP1), which localizes at the sites of DSB and forms nuclear radiation-induced foci (RIF) (11, 12). In irradiated cells, on DNA DSB sites, 53BP1 foci colocalize with Serine 139 phosphorylated histone H2AX foci (γ-H2AX) flanking a larger area around a DSB and hence considered another sensitive marker for DNA DSB damage (13, 14).

Combination of Cell-FIT-HD technology, clonogenic assay, and RIF detection should provide a platform for simultaneous analysis of microscopic beam parameters, particle effects on RIF formation and the ability of cells to form colonies as a function of particle number, quality, and spatial distribution.

## Materials and Methods

### Cell Culture

Cell lines used in this study were murine (Balb/c) renal adenocarcinoma cells (RENCA) and human alveolar adenocarcinoma cell line (A549), obtained from ATCC. RENCA were cultured in RPMI-1640 Medium (Gibco) supplemented with 10% fetal bovine serum (FBS, Gibco), non-essential amino acids (0.1 mM, Sigma), sodium pyruvate (1 mM, Sigma), and l-glutamine (2 mM, Sigma). A549 cells were cultured in Dulbecco’s Modified Eagle Medium (DMEM, ATCC) supplemented with 10% heat-inactivated FBS (Millipore), 2 mM glutamine, and 1% penicillin/streptomycin (complete DMEM).

### Cells Transduction and Immune Staining

A549 cells were transduced using a retroviral construct ­containing mCherry-53BP1-2 pLPC-Puro [Addgene plasmid # 19836; (15)]. Retrovirus production and cells transduction with mCherry-53BP1 construct were carried out, as previously described (15). Retrovirus production was performed using Retro-X Universal Packaging System (Clontech), according to manufacturer’s instructions. Transduction was conducted by the incubation of cells and viral particles in a complete medium containing 8 μg/ml Polybrene (Sigma) at 37°C, 5% CO_2_. Selection of transduced cells was performed using 2 μg/ml of Puromycin (Gibco). A549 cells expressing mCherry-53BP1 were cultured in complete DMEM containing 0.4 μg/ml of Puromycin (Gibco). All cells were incubated at 37°C at 5% CO_2_ atmosphere. A549 cells expressing mCherry-53BP1 construct were counterstained for γ-H2AX marker as described (16). Fixed (4% paraformaldehyde, for 10 min) and permeabilized (0.1% Triton-X for 10 min) cells were labeled using primary anti-γ-H2AX antibody (1:100, Cell Biolabs) and secondary Alexa Fluor 488-conjugated donkey ­anti-mouse antibody (Molecular Probes).

### Colony-Forming Cell Assay and Irradiation

For preparation of colony-forming cell assay using FNTD as a substrate (Cell-FIT-HD), FNTDs were first washed in an ultrasonic bath (Bandelin Sonorex) for 15 min at room temperature (RT). FNTDs were then placed in 70% ethanol overnight at RT. FNTDs were thoroughly washed in PBS, before used for cell culture.

Standard clonogenic assay (8) was performed using RENCA cells in six-well cell culture plates (200 cells/well). After attachment, cells were irradiated with ^12^C ion beam at Heidelberg Ion-Beam Therapy Center (HIT). Cells were positioned in the middle of a 1-cm widespread out Bragg peak (SOBP, 1 Gy) centered at approximately 3.5 cm water-equivalent depth, mimicking the clinical-like settings. Dose averaged linear energy transfer (LET) was 95 keV/μm. Non-irradiated cells were used as control. After colonies were formed, cells were fixed with 75% methanol and 25% acetic acid for 10 min at RT and stained with 0.1% crystal violet for 15 min.

Standard clonogenic assay was modified for studying the colony formation on FNTDs. Forty microliters of growth medium drop containing 50 cells were placed on the polished surface of the FNTD. The growth area was approximately 4 mm × 8 mm. For studying the ability of cells to grow on FNTD surface and form colonies, FNTDs containing cells (Cell-FIT-HD) were either irradiated as described above, or left without irradiation (control) and incubated for 7 days. After colony formation, cells were fixed and stained as in standard clonogenic assay (as above). FNTDs containing colonies were scanned (EPSON Scan). All obtained images were corrected for brightness and contrast by ImageJ (http://rsb.info.nih.gov/ij/) using the same image processing settings.

To correlate colony forming ability of a single cell and microscopic ion beam parameters, mCherry-53BP1 A549 cells were allowed to attach (100 cells/FNTD for control and 200 cells/FNTD for irradiated sample) at 37°C at 5% CO_2_ for at least 8 h prior to irradiation. Cell-FIT-HD was irradiated perpendicularly with respect to the incident ^12^C ion beam, as described above.

Approximately 30 min post-irradiation (*t* = 30 min), the entire area of the Cell-FIT-HD was imaged by widefield microscopy (see below). After the initial imaging, Cell-FIT-HD was placed in the incubator (37°C at 5% CO_2_ atmosphere) for 7 days to allow colony formation on the polished surface of the FNTD. The ability of colony formation with/without irradiation after 7 days (*t* = 7 days) was assessed by additional imaging of Cell-FIT-HD by widefield microscopy.

### Read-Out of Cell-FIT-HD

The read-outs of the physical compartment (FNTD) and of the biological compartment (single cells or colonies) of Cell-FIT-HD were uncoupled. 53BP1 (mCherry signal) and γ-H2AX (Alexa Fluor 488) in Figure 2 were imaged by Zeiss LSM710, Confocor 3 confocal laser scanning microscope, as previously described (3), at 30 min post-irradiation.

Initial cell attachment and colonies were imaged by the inverted widefield microscope Cell Observer (Carl Zeiss AG). To record the initial cell attachment, an overview scan of the entire cell attachment area (polished surface of the FNTD) was performed. Image stacks of regions of interests (ROIs) containing single cells were subsequently recorded. The stacks contained 41 and 45 image planes (each separated by 2 μm) for the imaging at *t* = 30 min, and at *t* = 7 days, respectively. The entire depth of the cell layer was covered. For each imaging plane, the bright field (BF) as well as the mCherry fluorescent channel (mPlum filter set) was recorded. After recording the overview scan, single ROI was subsequently imaged to allow for visualization of 53BP1 foci formation in individual cell nuclei of the colony. ROIs were chosen to match approximately the positions of time point 0. Individual tiles of the overview scan were corrected for shading and stitched using the ZEN software. The cells were washed away from FNTDs after the last widefield microscopy read-out. ROIs in the FNTD were then imaged by the Zeiss LSM710, Confocor 3 confocal laser scanning microscope. The imaging parameters were adjusted to gain optimal read-outs for the primary particles (5). The frequency distribution of fluorescence intensity of the ion tracks was assessed as a proxy for the LET spectrum. There are two distinct peaks that can be attributed to the primary carbon ions and the lighter fragments, respectively. A threshold was set to separate between the two species. Obviously, some heavier fragments might be considered as primaries, what, however, does not affect the generality of the results of this study. For each position, a *z*-stack of 35 imaging planes was recorded by 633 nm HeNe laser line (17). T-PMT detection was recorded in parallel. For widefield and for confocal imaging uncoated glass bottom, culture dishes (MatTek Corp.) were used.

### Registration of Biological and Physical Beam Data

Widefield (biological compartment) and confocal images (physical compartment) were registered employing point mapping to correlate cellular response to microscopic ion beam parameters spatially at time point 0. To this end, non-fluorescent Al–Al spinel cubical inclusions in the Al2O3:C, Mg crystal – both visible in the T-PMT and the BF channel – were used as point pairs. At least four point pairs were used yielding an accuracy of the projective registration smaller than 0.3 μm, i.e., on a sub-pixel scale. The same registration procedure was performed when projecting the nuclei positions at time point 0 into the cell layer at time point 7 days post-irradiation. It was ensured that the fluorescence (mCherry) and the brightfield channels of the widefield microscopy were spatially aligned.

### Ion-Hit Statistics

Due to perpendicular irradiation setup, all track spot centers at *z* = −3 μm (measuring from the FNTD surface, *z* = 0 μm) were projected onto the maximum intensity projection (MIP) of the 53BP1 mCherry signal of the cell layer. Positions of single ion traversals were assessed by using an in-house developed thresholding algorithm. To determine intranuclear ion hits, the positions of the track spot center (rounded to pixel values) were projected onto the nuclei mask of the MIP of the 53BP1 mCherry signal. Trajectory reconstruction and angle assessment confirmed the validity of perpendicular extrapolation (3, 5). In the imaging plane at approximately *z* = −3 μm each track spot was masked and the maximum intensity value assessed. The maximum intensity value was converted into count-rate and was corrected for non-linearity in APD detection (18).

## Results

### Colony Formation on FNTD

**Figure 1:**
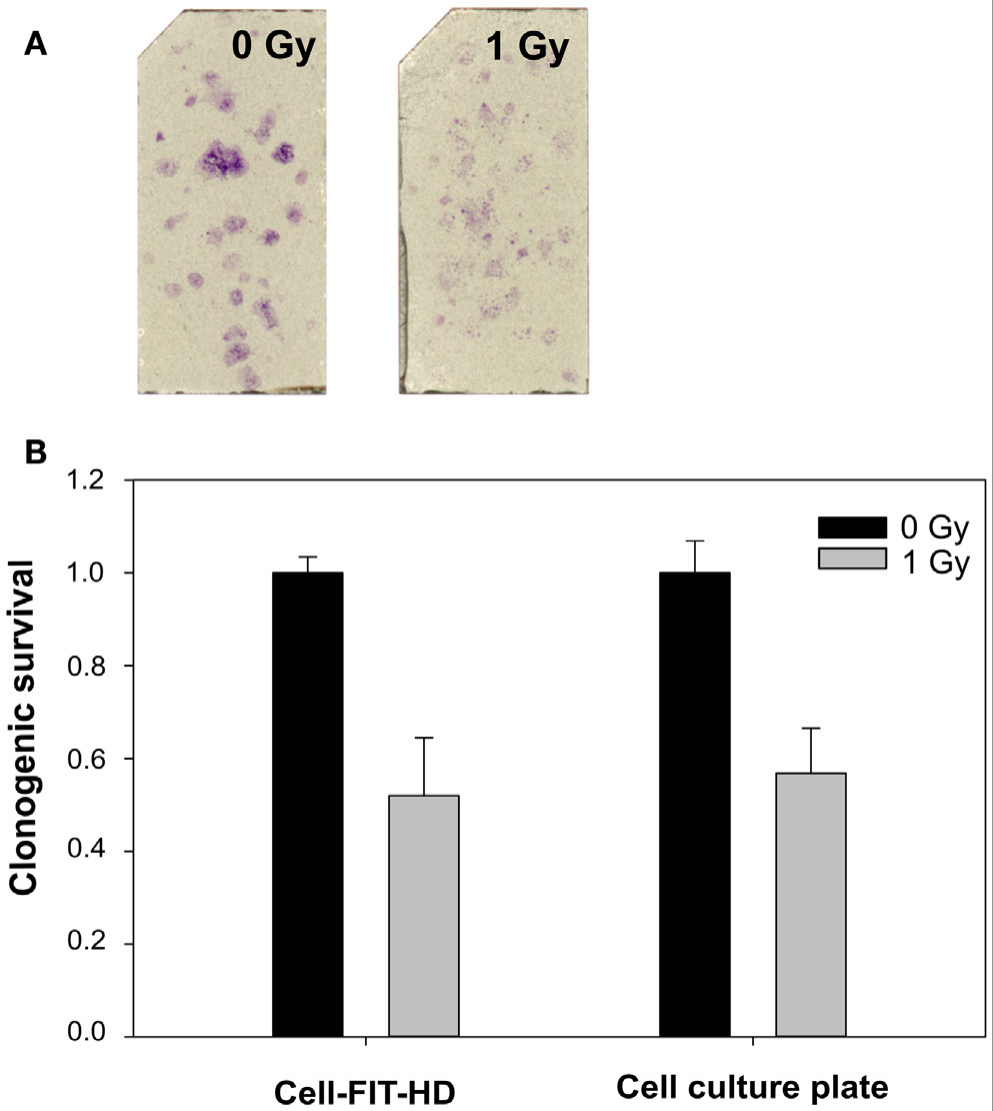
**Example of colony formation on FNTDs**. **(A)** Spots on FNTD surface: crystal violet staining of cell colonies. Brightness/contrast was adjusted for better visualization. **(B)** Comparison of clonogenic survival on FNTDs and in culture flasks. Means and SDs of triplicates are shown.

To study the feasibility of a FNTD’s surface for colony formation, murine renal adenocarcinoma cells (RENCA) were used. As shown in Figure 1A, the cells were able to attach and form colonies on FNTD surface. The mean plating efficacy and SD on FNTD surface was 33 ± 1.2%, whereas in a six-well plate it was 37 ± 6%. The results for colony formation and clonogenic survival on FNTDs correspond to those obtained using the standard clonogenic assay in cell culture dishes (Figure 1B).

**Figure 2:**
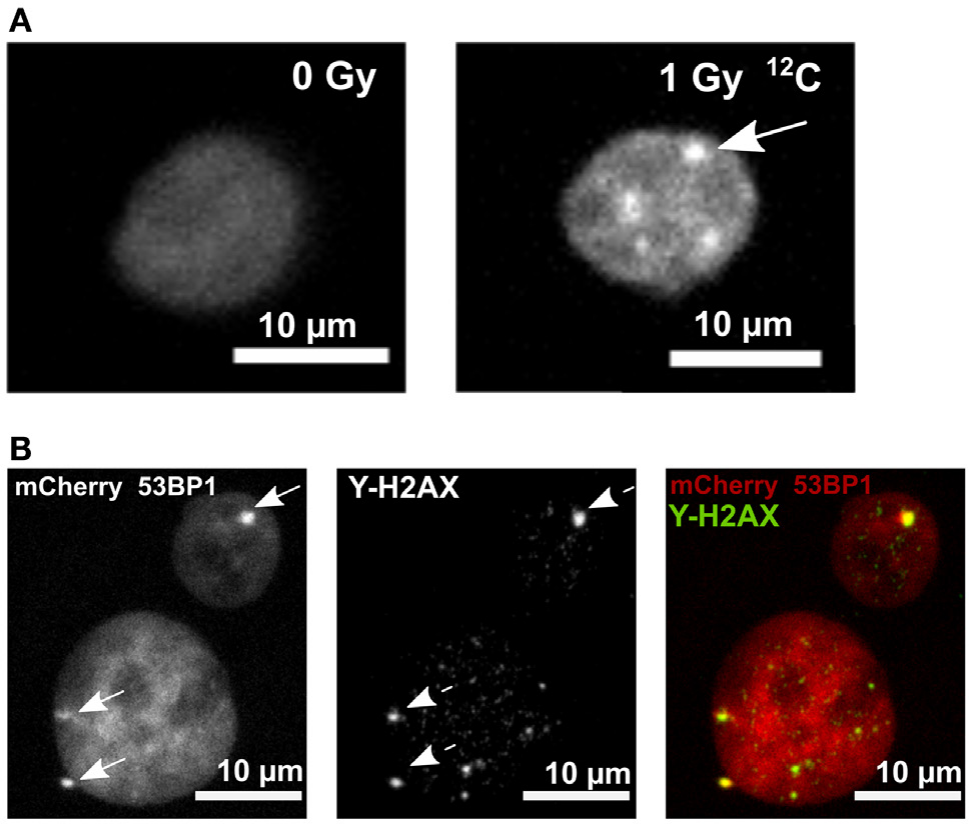
**mCherry 53BP1 and γ-H2AX signal in cell nuclei of A549 cells**. **(A)** Pan-nuclear expression of 53BP1-mCherry fusion protein in a control sample (panel left). Irradiated (1 Gy ^12^C) nucleus showing accumulation of 53BP1-mCherry signal (53BP1 foci, arrow). **(B)** mCherry-53BP1 signal (left panel, arrows point to 53BP1 foci), γ-H2AX signal (middle panel; dashed arrows point to γ-H2AX foci) in irradiated mCherry-53BP1 cells. Colocalization of 53BP1 and γ-H2AX foci (panel right). Sum of intensities of *Z*-stack slices is shown. Brightness and contrast were adjusted for better visualization.

To investigate colony formation on FNTDs, on a microscopic level, as well as DNA damage foci formation, we utilized human A549 cells expressing mCherry-53BP1 fusion protein. A549 cell line was selected because of its low level of background foci (2). The stable expression of the fluorescent fusion protein, localized in cell nuclei, provided homogeneous pan-nuclear staining, which enabled microscopic imaging of cellular nuclei, as well as individual foci formation after irradiation (Figure 2A). 53BP1 signal in irradiated cells colocalizes with γ-H2AX signal, which confirms the fact that 53BP1 accumulates at the DNA DSB sites (Figure 2B).

**Figure 3:**
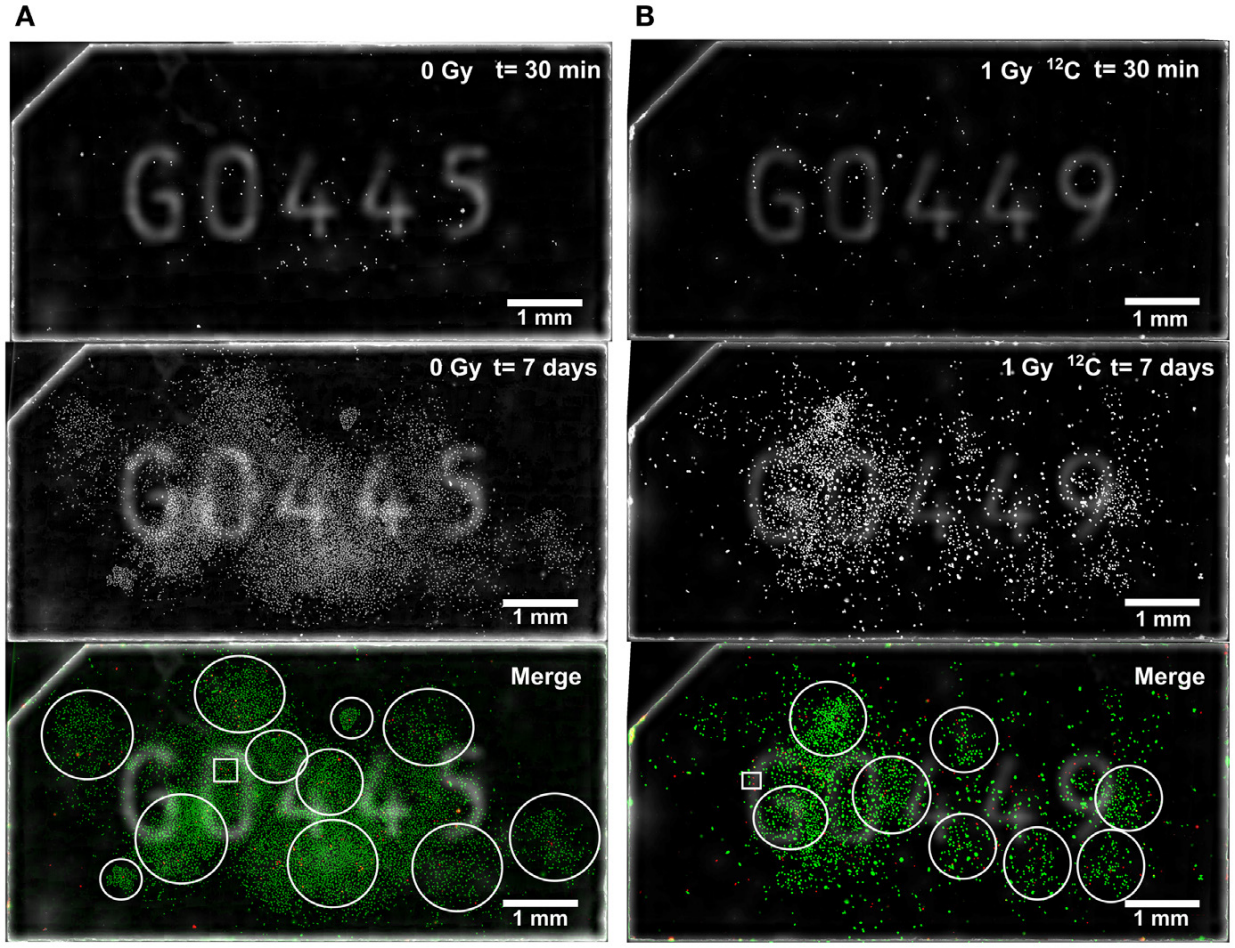
**Microscopic visualization of Cell-FIT-HD**. **(A)** Control sample (mock irradiation) and **(B)** irradiated sample (1Gy ^12^C-irradiation). FNTD surface was imaged at two time points: 30 min post irradiation (*t* = 30 min) and at 7 days (*t* = 7 days) post-irradiation. Early and late image orientations as well as brightness and contrast were adjusted and images were merged. Pan-nuclear mCherry-53BP1 signal was shown in red pseudocolor for *t* = 30 min, and in green pseudocolor for *t* = 7 days. White empty circles were used to mark different colonies. White empty squares indicate ROIs used for Figure 4. Numbers seen on FNTDs’ surface are identification numbers engraved in each FNTD.

In order to localize colony-initiating cell within a respective colony, the whole surface of Cell-FIT-HD was imaged at early (*t* = 30 min; red pseudocolor) and late (*t* = 7 days; green pseudocolor) time point, and the images were overlaid (Figure 3). At the seventh day post-irradiation, A549 cells formed dense colonies. This stands particularly true in case of control samples, where most of the cells were able to produce colonies (Figure 3A). Irradiated cells showed lower capability for clonogenic growth when compared to the control cells. They produced smaller colonies in comparison to a control sample, and many cells were not dividing (Figure 3B).

Even though cells can migrate on the surface during the colony formation time, it was assumed that the colony-initiating cell retained its position within the respective colony region. In the previous experiments, we observed that A549 cells can migrate up to 1 μm within 30 min in different directions, and these motion patterns of A549 cells would not be sufficient for a colony-initiating cell to leave the colony regions, especially in the case of larger colonies. Continuous live imaging of a colony formation was impossible due to the cytotoxicity induced by the long-term imaging settings.

### Irradiation-Induced Foci and Ion Hits

**Figure 4:**
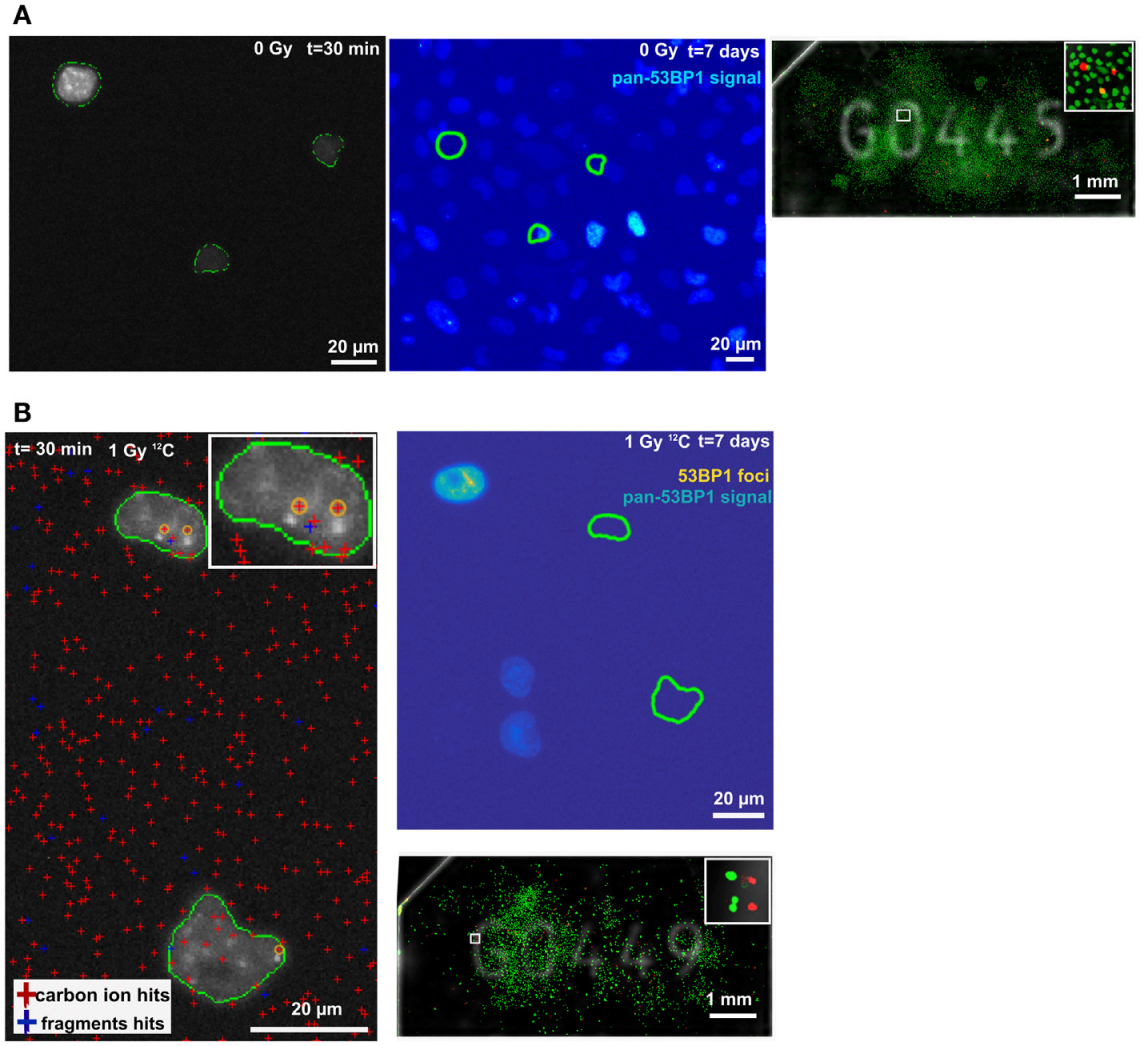
**Maximum intensity projections of 53BP1-mCherry signal and ion hits**. **(A)** Control sample at *t* = 30 min (panel left) and *t* = 7 days (large colony formation; middle panel). Position of selected ROI on FNTD (white empty square). Insert: magnification of selected ROI (panel right). **(B)** Irradiated sample at *t* = 30 min (panel left). The cross sectional area of the nuclei at *t* = 30 min is encircled in green. The bright spots in the nuclei are 53BP1-mCherry foci most probably induced by carbon ions (highlighted by yellow circles, closest proximity). The positions of ion traversals and fragments are indicated by the red and blue crosses, respectively. Insert: magnification of the upper nucleus. Upper right panel shows the irradiated nuclei at *t* = 7 days, and no colony formation. Dense aggregation of 53BP1 signal is marked in yellow. The position of the nuclei at *t* = 30 min is labeled by green lines. The positions were registered to *t* = 7 days using the unique spinel fingerprint of spinels in the FNTD. Position of selected ROI on FNTD (white empty square). Insert: magnification of selected ROI (bottom right).

To demonstrate the feasibility of a Cell-FIT-HD for analyzing ion traversals together with the irradiation-induced foci formation in a single cell, and investigate cell’s fate in regards to colony formation, ROIs were selected in both control and irradiated Cell-FIT-HD. For the control sample, ROI containing three cells (at the early time point) was selected. These cells divided multiple times forming a large colony (Figure 4A). Initial positions of the colony-initiating cells’ nuclei are marked by green closed lines (Figure 4A). At the seventh day post-irradiation, in case of irradiated samples, we analyzed the ROI containing cells that were not capable of colony formation (Figure 4B, right panel). Even though those cells did not form colonies, additional cells were found in their close proximity. This could imply either cell migration, or a single division of a cell (Figure 4B, top right panel). For the same ROI, we extracted the particle beam information from a physical compartment (FNTD) of a Cell-FIT-HD to visualize ion tracks. Within selected ROI, two nuclei showed large 53BP1 foci formation. Respective ion track spots were assigned to these irradiation-induced 53BP1 foci based on the closest proximity (orange circles, Figure 4B, left panel). These track spots were induced by primary-like carbon ions, since the imaging parameters were adjusted to detect primarily carbon ions. However, secondary high LET fragments can be in principle also included. Fast protons of low LET were not visualized. The ion beam fluency assessed was approximately 7.0 × 10^6^ particles/cm^2^.

## Discussion

Colony formation assay is a quantitative, macroscopic assay, which represents the standard for studying cell’s sensitivity to irradiation (8). It provides valuable information on the outcome of a large cell population upon the irradiation. However, this assay does not provide an insight on a single cell fate within a population, and why certain cells within a population will stop dividing and eventually die, whereas the other ones will still be capable of clonogenic growth. It can be hypothesized that certain cells within a population accumulate lethal level of irradiation-induced damage, and lose capability to divide and form colonies, whereas the other cells remain unaffected. The unaffected cells might have higher DNA damage repair potential or may be “missed” by the irradiation particles (19, 20). The first step for addressing these questions is to develop a platform that provides direct information about spatial distribution of irradiation particles and correlates it at a single cell level with the clonogenic capacity. For that purpose, we adapted the conventional approach for colony formation assay and combined it with the usage of FNTDs (Cell-FIT-HD). This approach enables simultaneous analysis of the microscopic beam parameters together with the events in colonies, single cells, and at sub-cellular level. We were able to show that single cells can attach and grow as colonies on FNTD surface. The size of a surface area of a FNTD (4 mm × 8 mm) is not optimal for clonogenic growth, resulting in many overlapping colonies, and therefore the current design of the Cell-FIT-HD is not suitable for performing large-scale quantitative clonogenic assay. This might be restricted to certain cell types, such as the RENCA, where circumscribed colonies could be detected at an early phase of colony formation. Further studies are needed to find the optimal constraints for colony forming cell lines/primary cells in the Cell-FIT-HD setting. Nevertheless, our primary purpose was the application for analyzing single colonies, single cells, subcellular structures, and microscopic beam parameters, which was successfully demonstrated.

The current work represents a proof of principle study for correlation of particle traversal with long-term colony formation using Cell-FIT-HD. The entire workflow is established and builds a solid foundation for further improvements toward population level quantitative analysis. Further application of Cell-FIT-HD may provide necessary information for dissecting underlying mechanisms for colony formation of irradiated cells, which is important for studying time-dependent repair capability analyzing eventually correlation between fast and slow repair and the complexity of the induced damage, as well as bystander effect.

## Author Contributions

ID, MN, FZ, AM, PS, and DK performed experimental design, acquisition, analysis, and interpretation of the results. They participated in manuscript writing, revision, and approval of the final version for submission and publishing. ID, MN, OJ, SG, JD, and AA participated in the conception and design of the work, as well as interpretation of the data. They participated in manuscript writing, revision, and approval of the final version for submission and publishing. All authors agree to be accountable for all aspects of the work in ensuring that questions related to the accuracy or integrity of any part of the work are appropriately investigated and resolved.

## Conflict of Interest Statement

The authors declare that the research was conducted in the absence of any commercial or financial relationships that could be construed as a potential conflict of interest.
